# Challenges of medicines management in the public and private sector under Ghana’s National Health Insurance Scheme – A qualitative study

**DOI:** 10.1186/s40545-016-0055-9

**Published:** 2016-02-24

**Authors:** Paul G. Ashigbie, Devine Azameti, Veronika J. Wirtz

**Affiliations:** Department of Global Health, Boston University School of Public Health, 801 Massachusetts Avenue, 3rd floor, Boston, Massachusetts 02118-2605 USA; Volta Regional Health Directorate, Ho, Volta Region Ghana

**Keywords:** Hospital, Insurance, Licensed chemical seller, Medicines, Pharmacy, Policy, Private, Public

## Abstract

**Background:**

Ghana established its National Health Insurance Scheme (NHIS) in 2003 with the goal of ensuring more equitable financing of health care to improve access to health services. This qualitative study examines the challenges and consequences of medicines management policies and practices under the NHIS as perceived by public and private service providers.

**Methods:**

This study was conducted in health facilities in the Eastern, Greater Accra and Volta regions of Ghana between July and August 2014. We interviewed 26 Key Informants (KIs) from a purposively selected sample of public and private sector providers (government and mission hospitals, private hospitals and private standalone pharmacies), pharmaceutical suppliers and NHIS district offices. Data was collected using semi-structured interview guides which covered facility accreditation, reimbursement practices, medicines selection, purchasing and pricing of medicines, and utilization of medicines. Codes for data analysis were developed based on the study questions and also in response to themes that emerged from the transcripts and notes.

**Results:**

Most KIs agreed that the introduction of the NHIS has increased access to and utilization of medicines by removing cost barriers for patients; however, some pointed out the increased utilization could also be corollary to moral hazard. Common concerns across all facilities were the delays in receiving NHIS reimbursements, and low reimbursement rates for medicines which result in providers asking patients to pay supplementary fees. KIs reported important differences between private and public sectors including weak separation of prescribing and dispensing and limited use of drugs and therapeutic committees in the private sector, the disproportionate effects of unfavorable reimbursement prices for medicines, and inadequate participation of the private sector providers (especially pharmacies and licensed chemical sellers) in the NHIS.

**Conclusions:**

Health providers generally perceive the NHIS to have had a largely positive impact on access to medicines. However, concerns remain about equity in access to medicines and the differences in quality of pharmaceutical care delivered by private and public providers. Routine monitoring of medicines use during the implementation of health insurance schemes is important to identify and address the potential consequences of medicines policies and practices under the scheme.

**Electronic supplementary material:**

The online version of this article (doi:10.1186/s40545-016-0055-9) contains supplementary material, which is available to authorized users.

## Background

In 2003, Ghana set out to provide Universal Health Coverage (UHC) to its citizens by establishing a National Health Insurance Scheme (NHIS). The World Health Organization (WHO) defines UHC as “access to key promotive, preventive, curative and rehabilitative health interventions for all at an affordable cost, thereby achieving equity in access” [1]. Medicines constitute an important component of health care interventions both in terms of cost to the health care system and their efficacy. The Forum on Universal Health Care Coverage held in Mexico City, further highlighted access to affordable, quality, safe and effective medicines as an important element of UHC [2]. The inclusion of UHC in the Sustainable Development Goals adopted by the United Nations in September 2015 widens a window of opportunity to improve access to quality and affordable medicines, especially in low and middle-income countries [3].

Ghana established the NHIS through a National Health Insurance Law (Act 650) in 2003 to ensure more equitable financing of health care and to improve access to health services in the country [4]. This law abolished the “cash and carry” system originally introduced in the 1980s. The National Health Insurance Act established a National Health Insurance Authority (NHIA) to regulate all health insurance schemes in the country and to implement the NHIS [4]. The NHIS is primarily financed by funds generated from a National Health Insurance Levy (a 2.5 % levy on goods and services collected under the Value Added Tax (VAT) system), 2.5 percentage points of formal sector employees’ monthly social security contributions, and premiums paid by subscribers in the informal sector [5, 6]. Formal sector employees and the self-employed who make social security contributions, children under the age of 18, individuals 70 years old and above, pregnant women, indigents, categories of differently-abled persons, persons with mental disorder, and social security pensioners do not pay membership premiums [7]. These groups constitute 69 % of active NHIS members. As of December 2013, active membership of the NHIS was 10.5 million representing 39 % of the total population. The benefit package of the NHIS covers 95 % of disease conditions in the country [5, 7].

A report published by the World Bank in 2013 indicated that more than 50 % of patients seek care from the private sector in Ghana [8]. Ghana has a private sector development policy that, among other goals, aims to build the capacity of private healthcare providers and increase access to private sector health services for the poor [9]. In line with this policy, the NHIA accredits both private and public health facilities to provide services covered by the NHIS. As of 2012 a total of 3,575 health service facilities had been accredited by the NHIS, including 1,916 public facilities, 866 private hospitals, and 207 mission facilities, as well as 324 pharmacies and 233 licensed chemical sellers (LCS) [10]. There are over 2,400 pharmacies and 10,000 LCS in the country [11]. Faith-based organizations (mission hospitals) constitute an important network of health care providers in Ghana. These providers tend to align more with the public sector as they benefit from government subsidies and their staff are normally on government payroll. The largest network of this group is the Christian Health Association of Ghana (CHAG).

Licensed chemical sellers and pharmacies tend to be first point of call for patients because there are no consultation fees, little or no waiting times and patients’ preference for self-medication [11]. LCS typically operate in rural areas, while pharmacies predominantly serve urban areas. LCS are limited by the Pharmacy Act of Ghana to selling only class C (over the counter) medicines (which account for about 30 % of total market value) [12]. It is likely, that insurance policies and practices differentially affects access to quality and affordable medicines at these facilities.

The NHIS has a periodically updated reimbursement list of medicines which the scheme covers [13]. As of 2014, this list has 522 medicines, which are provided to insured patients for free with no copayments [11, 13]. The NHIS determines fixed maximum reimbursement prices for medicines on the list, based on the median market price of each medicine [11]. Medicines are currently reimbursed on a fee-for-service basis, separately from other services, which are reimbursed under the Ghana Diagnosis-Related Group (GDRG) model. This reimbursement model enables standalone drug outlets to dispense medicines under coverage of the NHIS. Reimbursement prices for medicines covered by the NHIS is the same for public and private providers [11, 13].

The NHIS in Ghana is the subject of numerous studies, which generally focused on variations in health-seeking behavior based on insurance status. For example, in a cross-sectional household survey of 365 malaria patients, Fenny et al. showed that the patients with insurance are six times more likely to seek care from regional or district hospitals, five times more likely to seek care from health centers or clinics, and seven times more likely to seek care from private hospitals or private clinics as compared patients who are uninsured [14]. Few studies have focused on medicines and even fewer examine how the NHIS affects access to medicines through the private sector. Some reports have highlighted increases in total national expenditure on medicines, delays in medicines reimbursement, a decrease in the number of standalone pharmacies and LCS participating in the NHIS over time, and supplier-induced demand for medicines [11, 15, 16]. However it is unclear whether and how these findings differ between private and public institutions. This qualitative study examines the issues generated by the medicines management policies and practices introduced under Ghana’s NHIS as perceived by providers. In particular, it elucidates how these challenges and consequences differentially affect public and private service providers. The major pharmaceutical management practices the study focuses on include reimbursement strategies for medicines, medicines selection and use of formularies, purchasing and pricing, and medicines utilization.

## Methods

This study was conducted between July and August 2014 in the Greater Accra, Eastern and Volta regions of Ghana. Health facilities and institutions were purposively selected based on geographic accessibility, size (mix of big and small health facilities) and familiarity with the investigators. The facilities and institutions included public sector providers (regional, district and mission hospitals), private sector providers (private hospitals, and private standalone pharmacies), pharmaceutical suppliers and NHIS district offices. For the purpose of this study, we classified mission hospitals as “public” for reasons discussed in the background section.

Potential KIs were informed about the objectives of the study and invited to take part in the interviews. KIs were also asked to recommend other experts involved in medicines management in other health facilities for the study. In all, 26 KIs out of 29 agreed to participate and only three of these were referrals. Table 1 shows the number of KIs from each type of facility. Additional information on the type of facilities participating KIs worked is in Additional file 1.

**Table 1:** Number and type of facilities participating in each type of study

Type of institutions	Number of interviews	Interviewees’ Roles
Public hospitals (Regional, government or mission)	5	Heads of pharmacy
Private hospital	4	3 Medical Directors
		1 accountant
Private pharmacy	10	9 Superintendent Pharmacists
		1 Pharmacy Manager
District Insurance Office	2	1 District NHIS Manager
		1 District NHIS Public Relations Officer
Supplier	5	3 Chief Executive Officers
		2 Sales Executives
Total	26	

Interviews were conducted using a semi-structured interview guide to collect data on the experiences of KIs and their institutions managing medicines under the current health insurance policies and practices.

At the start of the interview, informed consent was solicited verbally to those who agreed to participate. The interview guide (Additional file 2) included questions about insurance policies and practices regarding: (1) facility accreditation, (2) reimbursement practices, (3) medicines selection and use of formularies, (4) purchasing and pricing, and (5) medicines utilization. These topics were adapted from the four strategies described by Faden et al. for balancing the competing goals of improving access to medicines, encouraging appropriate use, and keeping costs affordable [17]. Interviews lasted between 35 min to one hour. Fourteen interviews were tape-recorded with the consent of the KI. In the other cases where the interviewees refused to be tape-recorded, detailed notes were taken.

### Institutional Review Board Approval

The study protocol was reviewed and approved by Boston University’s Institutional Review Board.

### Data analysis

**Fig. 1:**
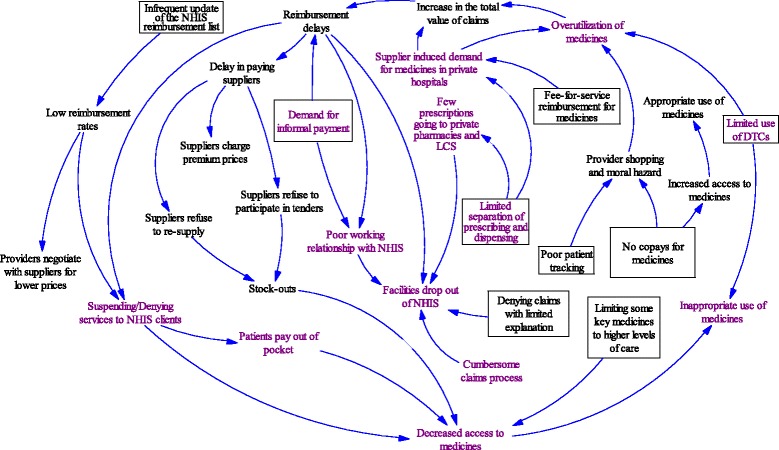
Diagram summarizing medicines policies and management practices and their consequences under Ghana’s NHIS. Legend: Policies and practices and independent attributes are presented in boxes. Consequences are presented in texts without boxes. Consequences and practices predominant in the private sector are colored in purple. The arrows show the potential inter-relationships among policies and practices, and their consequences

Interview recordings were transcribed and coded together with the interview notes. One of the investigators (PGA), who is experienced in qualitative data analysis developed codes based on the study questions and also in response to themes that emerged from the transcripts and notes. Responses from the public providers were compared with those from private providers. For the purposes of this analysis, we treated mission hospitals as “public” since their interaction with the NHIS system is similar to public facilities. We also explored variations within each provider type. Analysis focused on exploring both parallel and divergent perceptions. We explored the potential relationships among medicines management policies and practices and their consequences as reported by KIs. This is presented in a loop diagram in Fig. 1.

## Results

The results are presented according to the key areas of medicines management: 1) Accreditation process and discontinuation of service provision under the NHIS; 2) Reimbursement practices for public and private sector service provision; 3) Selecting medicines for purchase and use by facilities; 4) Purchasing and pricing of medicines; and 5) Improving medicines utilization.

1. **Accreditation process and discontinuation of service provision under the NHIS**

Most of the KIs reported that the accreditation process was easy and efficient. Below is a list of reasons given by KIs from private hospitals and pharmacies for applying for NHIS accreditation (It is obligatory for public facilities to provide services under the NHIS).

- To serve their community members who have health insurance
- To avoid losing their clients
- To make a profit
- To keep good working relationships with hospitals (including filling prescriptions coming out of hospitals)
- To become part of the main health care system

Anecdotal reports suggest that facilities (especially private facilities) accredited by NHIS often drop out after an initial period. All KIs in the public sector said it is difficult for public providers to drop out of the NHIS or suspend services to NHIS patients. In contrast, all KIs from pharmacies and private hospitals shared the perception that dropping out of the NHIS was quite common among their peers, although one mentioned it was not common *in his area*. Providers gave multiple reasons for dropping out of the NHIS. These reasons, some of which were corroborated by KIs from the NHIS include delays in reimbursement, unfavorable reimbursement prices for medicines, not having enough business from NHIS-affiliated patients, having enough patients who can afford to pay for services out of pocket, the extensive labor involved in processing claims, NHIS refusing to reimburse for medicines dispensed on suspicion that the claim was fraudulent, and poor relationships with NHIS staff who sometimes demand informal payments. KIs from two NHIS district offices confirmed the limited participation of pharmacies and LCS in the NHIS. Only one LCS (who had stopped serving NHIS clients at the time of the study) have been accredited in these two districts. The following quotes illustrate some of the reasons providers drop out of the NHIS:

“*Because it is not a big business, we do not want to set up structures for it. We only receive 3–5 prescriptions in a month.*” – KI P002, Private Pharmacy

*“They [NHIS] frustrate you for so many reasons […]. Once you do not give them kickback they delay the vetting and submission of your claims. For the public sector they know they cannot collect any money from them, so the focus is on the private sector.”* – KI PH001, Private Hospital

Instead of officially opting out of the scheme, some reported other ways of avoiding NHIS clients:

*“Sometimes we tell [NHIS] patients we do not have the medicine. Though we have it, we prefer selling it to individuals who can pay.”* – KI P007, Private Pharmacy

Two KIs from CHAG facilities said that most CHAG institutions have decided not to provide services under NHIS, under the direction of their leadership. This decision (which coincided with our data collection period) was due to facilities being owed seven or more months’ arrears by NHIS, the high market prices of medicines compared to the NHIS reimbursement prices, and the rapid depreciation of the cedi, which results in facilities incurring more cost if they do not get timely reimbursements.

Another KI from a CHAG facility gave an example of how their facility is trying to strike the right balance between suspending services and taking care of the vulnerable:

*“… For pregnant women we were giving them all the routine drugs, paracetamol and malaria treatment. But for antibiotics unfortunately they have to pay, using the wards they have to pay [out of pocket]….That is what we are practicing up to today.*” KI PU007, Mission Hospital

2. **Impact of reimbursement practices on public and private sector service provision**

A majority of KIs from pharmacies (eight out of ten) and all of the KIs from private hospitals felt it was not fair that the reimbursement prices for medicines are the same for both the public and private sector providers. They felt that private providers incur additional expenses, including payment of salaries, utilities, and taxes, which are paid by the government for the public sector. However, KIs from the NHIS said they have not received any official complaint about the need to have higher reimbursement rates for private providers.

All KIs in both public and private sectors as well as three (out of five) suppliers reported the NHIS medicine reimbursement prices often fall below market prices and the prices are not regularly updated. Some participants blamed higher prices for medicines on a recently-introduced government policy to charge VAT on medicines. This tax has since been removed.

Constrained by low reimbursement rates, providers face a dilemma: do they dispense medicines at the current NHIS price and incur losses, or do they ask patients to pay the difference? One KI said the latter option has been discouraged by the NHIA. However comments from other KIs, including an NHIS staff show that this does happen:

*“If the price is high, and the person wants the drug, he may pay the difference. We call it ‘top-up’.”* – KI PH004, Private Hospital.

*“Some co-payment [for medicines] exist, but they are not permitted by the NHIS” –* KI N001, NHIS

Another strategy used by providers to circumvent low reimbursement rates for specific medicines is prescribing a therapeutically equivalent alternative that has a higher mark-up. For example, the reimbursement price of amoxicillin capsules is below the supplier price. When patients present with simple respiratory tract infections, providers prefer prescribing amoxicillin/clavulanic acid (a more expensive treatment) instead of amoxicillin only (which is less expensive).

### Consequences of delays in reimbursement

All of the providers, private and public, complained about reimbursement delays (in some cases up to nine months) which is also affects their ability to pay suppliers on time. Suppliers confirmed that delays in reimbursement to facilities results in delays in getting paid for their supplies. Accredited facilities, they noted, take longer to pay compared to facilities that are not accredited by the NHIS.

Suppliers mentioned various strategies they use to force facilities to pay them in time. These include: charging a premium price for medicines supplied to NHIS-accredited facilities to offset delays (mentioned by one supplier); lobbying facilities to get the NHIS to pay them in time (two suppliers); approaching the facilities immediately when they learn that the NHIS has paid them (one supplier); and reducing supplies to the facility or refusing to supply or participate in tenders altogether (four suppliers).

3. **Selecting medicines for purchase and use at health facilities**

KIs from both the private and public sector said that they use the national Essential Medicine List (EML), Standard Treatment Guidelines (STG), and the reimbursement list when deciding which medicines to stock at their facilities. All KIs concurred that the reimbursement list has become more important, though the STGs are used as treatment protocols and for audit purposes. All of the public facilities reported stocking, based on need, a few medicines outside the reimbursement list and/or EML that they sell on a cash and carry basis.

An important difference exists between private and public hospitals: the presence of drugs and therapeutics committees (DTCs). All KIs from public hospitals reported having DTCs in their facilities; in contrast, DTCs are not generally available in private institutions. Only one KI from a big private hospital reported having a DTC. Facilities mentioned their DTCs guide the selection of medicines and approve the use of medicines outside the EML and reimbursement list; advise on rational use of medicines; develop an institutional formulary; and disseminate information on clinical claims management.

4. **Purchasing and pricing of medicines**

### Purchasing of medicines

Public and private sector providers utilize different procurement strategies. Government hospitals are required to use the Central Medical Stores (CMS)/Regional Medical Stores (RMS) as their first supply source. Faith-based organizations (mostly Catholic hospitals and clinics) have another source in addition to the CMS and RMS; they source products from the National Catholic Secretariat (NCS), which does pooled procurement for affiliated facilities. The CMS, RMS, and NCS procure large volumes of medicines by tender.

KIs in the private sector, on the other hand, reported they are not able to purchase by competitive tender (due to the small volumes they procure). Instead, they compare price lists from suppliers for the best price, sometimes negotiating with suppliers using the reimbursement list. Table 2 summarizes the advantages the KIs reported for competitive tender and negotiation or direct procurement. Suppliers acknowledged that they may reduce prices during competitive tenders

**Table 2:** Advantages of competitive tender versus direct procurement reported by KIs

Competitive tender	Competitive negotiation/Direct procurement
-Lower procurement prices (reported by 17 KIs)	-Less expensive process (reported by 1KI)
-Assurance of getting quality medicines (6 KIs)	-Wider pool of suppliers to select from (1KI)
	-Most suppliers deliver medicines to providers for free (1 KI)

*“[Public facilities providing] NHIS purchase by competitive tender. We tend to come down a little on their prices. In fact, it should have been the opposite since they will not pay you on time, but because of tender we give them lower prices.”* KI S004, Supplier.

While one private sector KI mentioned private providers are not entirely barred from accessing medicines through the CMS and RMS, others highlighted major difficulties in getting medicines from the public medical stores. Sales by the CMS/RMS to private providers are cash and carry only, and the CMS/RMS prioritizes public facilities over private facilities.

### Pricing of medicines

Mark-ups applied to medicines varied widely between and within public and private sector providers, as well as across medicines. All KIs from suppliers said they are guided by the NHIS reimbursement list when determining their selling prices. Suppliers reported mark-ups ranging on average between 10 and 40 %.

At the retail level, private hospitals generally reported adding a mark-up of 25 to 40 % for medicines that are not on the reimbursement list (The exception was one KI who said he adds 1 or 2 %, but this was stated unsurely). Private pharmacies reported using mark ups between 30 and 50 % for the same category of medicines depending on the location of the facility (facilities in affluent neighborhoods had higher mark-ups), the price of the medicine (expensive medicines had lower mark-ups), demand for the product (products with low demand had higher mark-ups), and making financial transactions easier (rounding up mark ups to the nearest round amount). Public facilities reported mark-ups ranging from 15 to 40 % for medicines not on the reimbursement list, which their patients pay for out of pocket.

5. **Improving medicines utilization**

Almost all KIs (including the two KIs from the NHIS) felt that the NHIS has led to increased utilization of medicines, reporting: patients who could not afford to purchase medicines now have free access to them; and, the NHIS has enabled patients to stop purchasing incomplete doses of medicines, especially antibiotics.

KIs from both the private pharmacies and the public hospitals also mentioned negative impacts: over-prescribing by those providers who also dispense medicines or have pharmacies in their facilities; and “provider shopping” by patients. Because there is no centralized tracking system, patients go from facility to facility with the same complaint, soliciting medicines, which according to KIs, they try to resell on the black market. Thus some negative impacts are provider-driven, while others are patient-driven.

*“Some clients assume they must by all means benefit, so you find a few of the clients going from one facility to the other to access the medications – without finishing their previous medications*” KI PU001, Public Hospital

NHIS does not reimburse medicines dispensed from a facility below the level of care that medicine is assigned in the EML. While the purpose of this is to promote appropriate use of medicines, three KIs working in the private sector observed that this has a negative impact on access to some of these particular medicines. Patients may have to travel long distances or wait to get to a referral facility to access these medicines, including medicines that could be given to stabilize patients before referral.

All KIs from private pharmacies and public hospitals reported that it is common in private hospitals for prescribers to dispense or have influence over the activities of those dispensing. This goes against the recognized best practice of clearly separating the roles of prescribing and dispensing (This was not seen to occur in the public sector). KIs from private pharmacies expressed concerns about how this affects pharmaceutical care and how prescriptions from private hospitals do not get to private pharmacies.

*“Physicians prescribe what they can dispense, not what the patient needs….Pharmacy practice is now just a practice without patients.”* – KI P006, Private Pharmacy

Two KIs from private hospitals however asserted that there is separation of prescribing and dispensing in private hospitals. One mentioned this cautiously, noting that the NHIS requires the hospital pharmacy to be manned by a dispensing technician or pharmacist, but this is difficult because of lack of human resources.

KIs were asked whether government programs that seek to promote the appropriate use of medicines also reach the private sector. Eleven KIs (five from private pharmacies, and three each from private and public hospitals) agreed that the private sector is sometimes involved in these initiatives. Two KIs from private pharmacies further observed, however, that it is not mandatory for private sector providers to participate, unlike the public sector. Private hospitals mentioned receiving technical assistance without specifying if this assistance relates to medicines.

6. **Interrelationships among management policies and practices and their consequences**

Figure 1 presents a summary of the policies and practices examined in this study, their consequences reported by KIs, and the potential relationships among these. The figure also highlights how the consequences of these policies and practices differentially affect public and private sectors. The text presented in boxes describe policies and practices and independent attributes. The text presented without boxes show the consequences of these policies and practices. Consequences, policies and practices predominant among private sector providers are colored in purple in the figure.

## Discussion

The experiences revealed in this study show that, despite the overall strides Ghana has made towards universal health care coverage and improved access to medicines, critical challenges relating to access and use of medicines under the NHIS persist. In the sections below, we first discuss general cross-cutting themes that emanated from the study. Following this, we highlight important differences in the consequences of medicines management policies between private and public providers.

### Cross cutting themes

Most of those interviewed agreed that the introduction of the NHIS has increased access and utilization of medicines, due to removal of cost barriers for patients. This concurs with findings from other studies, including those that have documented Ghana’s progress towards achieving Millennium Development Goal 8E, which aimed to provide access to affordable essential medicines in developing countries [18–20]. The importance of the financial protection provided by UHC in enhancing access to medicines is documented. A 2007 to 2010 household survey on access to medicines for chronic diseases in five low- and middle-income countries, including Ghana, showed that those who are insured are up to three times more likely to have access medicines for chronic diseases, compared to those who are uninsured [21]. Increasing access and utilization bears the risk of inappropriate use if medicines are not managed adequately. For example, a study by Witter et al. on the NHIS found that medicines utilization, measured by number of medicines per prescription, increased from 4.5 in 2004 to 6.0 in 2008 [22]. The WHO and the International Network for Rational Use of Medicines recommends an optimum average number of medicines prescribed per consultation of ≤3 [23, 24]. This recommended figure is less than half that of Ghana’s in 2008. Increased utilization could also be due to moral hazard, where patients frequently use services that they do not need because they do not have to pay for it. While the absence of copayments for medicines under the NHIS will remove financial barriers to access to medicines, this very access also creates opportunities for overutilization of medicines [17]. Increased utilization of medicines may also be due to supplier- or prescriber-induced demand; this can be exacerbated by limited separation of prescribing and dispensing, especially in the private sector [15]. Increase utilization in the absence of any clinical need could harm patients and –in the case of anti-infective medication- result in the development of antimicrobial resistance.

The NHIS reimbursement list appears to be accomplishing its purpose - streamlining purchases and containing costs - as suppliers reported using it as a guide when pricing their products and providers reported taking it into account when bargaining for or purchasing medicines. Thus the reimbursement list has the potential to control the price of medicines. There is limited documented evidence on the effects of formularies in reducing medicines prices in low and middle income countries [17]. The use of formularies has been shown in some places to decrease the use of imported and expensive medicines, for example, reducing the rate of growth for total medical and medicines expenditures in China and Taiwan [25, 26]. A balance must be struck between cost containment on the one hand, and the sustainability of health facilities and industry on the other, to ensure reliable availability of medicines. The practice of asking patients to pay supplementary fees to make up for low reimbursement prices (despite being discouraged by the NHIS) presents an opportunity for providers to charge arbitrary prices. While this allows the profit making inherent in the private sector, it may interfere with equity in access. Additionally, it threatens the goal of the NHIS to provide financial protection to patients.

Another common concern reported across all facilities was the delay, up to 7 to 9 months, in receiving reimbursements from NHIS. This has also been reported by other studies [11, 27, 28]. As shown in Fig. 1, these delays affect both the providers and the suppliers. In response, suppliers adopt strategies such as refusing to re-supply providers, patients ultimately suffer from being denied treatment due to financial barriers or stock-outs.

Loop diagrams have been used to show complex relationships among health systems policies and practices [29, 30]. As shown in the loop diagram in Fig. 1, the web of interrelationships among medicines policies and management practices and their consequences can be complex. A policy or practice could have unintended and negative consequences in addition to its desired effects. For example, not having copays for medicines will improve access to and promote rational use of medicines. However, this policy can also promote overutilization of medicines and a concomitant increase in the value of claims. The sustainability of the scheme can be jeopardized as claims reimbursement delay and facilities drop out of the scheme. It is thus important in the design of insurance schemes, to have a system of monitoring and addressing unintended consequences of medicines policies and management practices.

### Major differences between private and public providers

#### Participation of providers

Though KIs from both private and public sectors were satisfied with the process by which NHIA accredits health facilities, there is limited participation of LCSs and private pharmacies. At the time of the study, no LCSs or private pharmacies were providing services under NHIS coverage in two districts we visited. Private providers are more adversely affected by the factors that discourage NHIS participation (Fig. 1). Delays in reimbursement, unfavorable reimbursement prices for medicines, and the labor involved in processing claims likely have more impact on private sector providers, especially LCS, private pharmacies and small hospitals, which operate with little capital and low human resource capacity. These explain why it is more common for private facilities to drop out of the NHIS and deny NHIS patients treatment. This does not auger well for Ghana’s private sector engagement policy. Considering the large proportion of private providers in the country, their retention in the NHIS program is important in improving access to medicines.

#### Separation of prescribing and dispensing

Separation of dispending and prescribing functions may be relatively poor in the private sector. As shown in Fig. 1, the limited separation of prescription and dispensing, coupled with fee-for-service reimbursement for medicines, may create an environment for supplier-induced demand. This in turn leads to increased use of higher cost, inappropriate medicines and limited prescriptions reaching private pharmacies and LCS. In Zimbabwe, prescriptions by dispensing doctors include about twice the number of medicines when compared to prescriptions from non-dispensing doctors [31]. An even more extreme example come from Malaysia, where dispensing doctors were documented to prescribe seven times more medicines than non-dispensing doctors [32, 33]. Separating the roles of prescribing and dispensing limits over-prescription by dispensing doctors and incentives for inappropriate selection of medicines [33].

Protecting professional boundaries has been reported as a barrier to separating prescribing from dispensing [34]. KIs in this study also mentioned limited human resources as a barrier – there may not be enough personnel available to divide the roles. Despite these challenges, separation is necessary in the delivery of quality pharmaceutical services.

#### Use of drug and therapeutic committees

The absence of DTCs in private hospitals should be of concern to policy makers. DTCs have been shown to contribute to optimizing patient health outcomes, controlling expenditure, managing formularies effectively, and educating health workers on medicines [35–41]. The frequent absence of DTCs in private hospitals presents a situation of inadequate oversight of medicines use in this sector. As patients now have equal opportunities, depending on their location and preference to seek services from the public sector and accredited private providers, implementing DTCs in the private hospitals is necessary to promote rational use in all sectors.

#### Purchasing

Public facilities reported purchasing by tender or sourcing their medicines from the medical stores, which purchase in high volumes and therefore at lower prices. Private facilities more often engaged in negotiated or direct procurement, often in smaller volumes. Generally, bulk purchasing and competitive tenders could lead to lowered prices. For example, bulk purchasing of selected essential medicines saved the government of Delhi, India, 30 % of its annual medicines expenditure [42]. In another case, six small islands in the South Pacific jointly employed this strategy to achieve the economies of scale [43]. Private providers in Ghana are likely confronting higher procurement prices due to their fractured purchasing. Supporting private providers to purchase products from medical stores would ensure they have access to better prices and higher quality medicines (as the medical stores are likely to have more technical expertise and resources to ascertain medicines quality). This could also benefit the medical stores by increasing their customer base and negotiating and purchasing power. However, they might need to adapt some of their practices to meet the particular requirements of the private sector.

There are some limitations to this study. Due to the centralized governance system in Ghana, we expect our findings from the three regions to be representative of the country. However, extrapolating the findings of this study to other countries need to be done with caution as the health systems structure and organization, culture, and policies may be different. Additionally, we could not get a KI from LCS to participate in the study. Even though LCS perform the same role as private pharmacies, we acknowledge our findings might have missed some of their perceptions.

## Conclusion

The findings from this study suggest that there are positive and negative consequences of medicines management policies and practices under Ghana’s NHIS. In addition to confirming findings from previous studies of Ghana’s NHIS, this study provides new insight on how medicines management policies and practices differentially affect public and private providers. Delays in reimbursement and low reimbursement prices have been identified as particular challenges that affect private sector providers more and have led to their limited participation in the scheme. Additionally, there may be inadequate oversight of medicines use in the private sector due to the lack of separation between prescribing and dispensing and the limited use of DTCs. Based on these findings, the following are some options to consider to improve the quality of pharmaceutical care under the NHIS.

1. Implement a routine system for monitoring and evaluating the effects of insurance policies on medicines use in the public and private sectors.
2. Frequently update reimbursement prices to reflect market prices. It may also be worthwhile to have reimbursement rates that are reasonably different between public and private providers.
3. While the limited human resource in the country will not make separation of the role of prescribing and dispensing possible in all private hospitals, the concept should be promoted in big private hospitals. This should be made one of the criteria for upgrading facilities to higher levels of care.
4. The use of DTCs should be promoted in private hospitals. The existence of a DTC should also be one of the indicators for upgrading health facilities to higher levels of care.
5. Private hospitals should be allowed to purchase from CMS and RMS if they chose to, so they have access to quality products and potentially better prices.

Equitable access to quality and affordable medicines should continue to be a key consideration when designing both insurance policies and national accreditation processes. Recognition of their possible consequences on the various categories of providers including the private and public sector requires additional attention and assessment.

## Additional files

Additional file 1: Annex 1. Background of facilities of KIs interviewed (DOC 29 kb) Additional file 2: Annex 2. Interview guide for key informants. This document is the interview guide used to collect data from private providers, public providers, suppliers and officials of the National Health Insurance Scheme. (PDF 260 kb)
