# Supplementary material for: Challenges of medicines management in the public and private sector under Ghana’s National Health Insurance Scheme – A qualitative study
Source: J Pharm Policy Pract. 2016 Feb 24;9:6. doi: 10.1186/s40545-016-0055-9 (PMC4765108; doi:10.1186/s40545-016-0055-9)
Supplement: Additional file 1: Annex 1. — Background of facilities of KIs interviewed (DOC 29 kb) [file 40545_2016_55_MOESM1_ESM.doc]

***Annex 1:*** ***Background of facilities of KIs interviewed***

| *Type of facility* | *Background* |
| --- | --- |
| Private pharmacies | - All 10 had at some point in their practice been registered with the NHIS. - 1 has never provided service for NHIS clients since its accreditation in 2008. - 3 have officially dropped out of the scheme - 4 are in the scheme but have decided not to provide services to NHIS clients. - 3 are currently providing services under NHIS coverage. |
| Private hospitals | - Majority of their clients sought care under NHIS coverage. - Two hospitals estimate at least 90% of their clients were NHIS card holders. |
| Public hospitals | - 3 were government owned hospitals. - 2 were faith based institutions (one serves as a district level facility and other a clinic) |
| Suppliers | - All five suppliers had been supplying both over the counter and prescription only medicines for more than 10 years. - Two of these also manufacturer their own medicines. - Their clientele included both public and private sector providers - private pharmacies, licensed chemical sellers, public and private hospitals. - 2 of the suppliers reported 60-80% of their sales go to the public sector while 20-40% go to the private sector. |
