# Supplementary material for: Challenges of medicines management in the public and private sector under Ghana’s National Health Insurance Scheme – A qualitative study
Source: J Pharm Policy Pract. 2016 Feb 24;9:6. doi: 10.1186/s40545-016-0055-9 (PMC4765108; doi:10.1186/s40545-016-0055-9)
Supplement: Additional file 2: Annex 2. — Interview guide for key informants. This document is the interview guide used to collect data from private providers, public providers, suppliers and officials of the National Health Insurance Scheme. (PDF 260 kb) [file 40545_2016_55_MOESM2_ESM.pdf]

Annex 2 – The role of medicines in universal health care coverage

Interview guides

1. Interview guide for private and public sector service providers

Participant ID:
Sector (private or public):
Type of facility: (hospital, private pharmacy, licensed chemical seller)
Date of interview: ____/____/_____
Duration of interview (from/to):

**Contracting providers**
1. What are the common reasons private institutions would like to get accredited with the NHIS?
2. What are the processes of obtaining accreditation?
3. What challenges do providers face in obtaining accreditation?
4. How does the accreditation process differ between the different types of providers: private pharmacies, licensed chemical sellers, private hospitals and public hospitals?
5. Can you tell us about the number (and types) of facilities opting out of NHIS since 2004
6. What are the most common reasons private facilities opt out of the NHIS
7. Currently NHIS reimbursement is based on whether a medicine is on the NHIS list or not. Reimbursement rates are the same for both private and public providers. What have been the consequences (positive and negative) of this practice on the NHIS from the perspective of private institutions relative to public institutions?
8. To what extent do private hospitals use the National Essential Medicines List and Standard Treatment Guidelines in the selection of medicines they procure and purchase? Are these institutions targeted in the implementation of these documents?

9. To what extent do public hospitals use the National Essential Medicines List and Standard Treatment Guidelines in the selection of medicines they procure and purchase? Are these institutions targeted in the implementation of these documents?
10. To what extent do private hospitals use the National Health Insurance (NHIS) List of Medicines and the Standard Treatment Guidelines in the selection of medicines they procure and prescribe?
11. To what extent do public hospitals use the National Health Insurance (NHIS) List of Medicines and the Standard Treatment Guidelines in the selection of medicines they procure and prescribe?
12. Public hospitals are required to have drugs and therapeutic committees (DTCs). Does this apply to the private hospitals as well? What has been the consequences (positive and negative) of DTCs in both sectors?
13. What are some of the challenges faced in selecting medicines for use at the institutional level? How do these differ among public hospitals, private hospitals, private pharmacies and Licensed Chemical Sellers (LCS).

**Pricing and Purchasing**
14. What types of procurement strategies exist in private and public health facilities (open tender, restrictive tender, competitive negotiation, direct procurement from a single supplier etc).
15. What are the consequences (or possible consequences) of these procurement systems on the buyer (cost) price and quality of medicines available in private sector?
16. What pricing strategies exist for medicines **not** covered by NHIS in private and public institutions (cost plus (fixed margins), competitive pricing, value-based pricing?
17. How do reimbursement prices for medicines differ for both public and private health institutions? What has been the impact of the similar or difference reimbursement prices?
18. How does the NHIA determine the prices of the medicines on its list?
19. How often is the NHIA list of medicines revised or updated?

20. How do NHIS medicines prices compare to normal market prices?

Utilization of medicines
21. What has been the effect of the introduction of the NHIS on the utilization of medicines?
22. How is the separation of the role prescribing and dispensing enforced within the existing structures of the NHIS in private and public hospitals.
**23.** What has been the role of the private and public sectors in implementing the National Strategy for Containing Antimicrobial Resistance

Information Sources
24. What types of information sources do the government, the NHIS or Private Practitioners use in monitoring medicines selection, procurement, pricing and utilization for decision making purposes
25. What are the challenges with these information sources?
26. In using available information for policy making, what information gaps currently exist?
**Other consequences of the NHIS relating to medicines**
Are there other consequences of medicines management policies and practices under Ghana's NHIS you would like to share?

2. Interview guide for suppliers

Participant ID:	
Type of facility: (importer, manufacturer or both)	
Date of interview:	____/____/_____
Duration of interview (from/to):	

1. How do you use the NHIS medicines list, and the National Essential Medicines List in making decisions regarding the importation or manufacturing of your products
2. What medicines pricing strategies do you have for NHIS accredited and non-NHIS accredited health facilities?
3. What procurement strategies exist among public and private health care providers in the country? What are the consequences of these procurement strategies?
4. Are there any challenges you face supplying public and private providers? Do these challenges differ between the two providers?
5. Are there any challenges you face supplying NHIS accredited and non-NHIS accredited providers? Do these challenges differ between the two providers?
6. What measures to you take when facilities delay in payment for suppliers?
7. Can you tell us how prices on the NHIS list are determined? How often is the list updated? How do you use the NHIS list when pricing your products?
8. How do the NHIS prices compare to normal market prices?
9. What has been the effect of the NHIS on utilization of medicines?

10. How is the separation of the roles of prescribing and dispending enforced within the existing structures of the NHIS
11. Are there other consequences of medicines management policies and practices under Ghana's NHIS you would like to share?

3. **Interview guide for NHIS staff**

Participant ID:	
Date of interview:	____/____/_____
Duration of interview (from/to):	

1. What are the common reasons private institutions would like to get accredited with the NHIS?
2. What are the processes of obtaining accreditation?
3. What challenges do providers face in obtaining accreditation? And what challenges does the NHIS face in issuing accreditation to providers?
4. How does the accreditation process differ between the different types of providers: private pharmacies, licensed chemical sellers, private hospitals and public hospitals?
5. Can you tell us about the number (and types) of facilities dropping out of NHIS since 2004? Is there any formal process for dropping out of the scheme?
6. What are the most common reasons private facilities drop out of the NHIS or decide not to provide services under NHIS coverage?
7. What have been the consequences of having the same medicines reimbursement rates private and public sector providers?
8. How is the NHIS list revised? Any challenges revising the list? How often is the list revised?
9. How do NHIS medicines reimbursement prices compare to normal market prices?
10. What has been the effect of NHIS on medicines use?

11. Are there other consequences of medicines management policies and practices under Ghana's NHIS you would like to share?
